# Impact of pain and postoperative complications on patient-reported outcome measures 5 years after microvascular decompression or partial sensory rhizotomy for trigeminal neuralgia

**DOI:** 10.1007/s00701-017-3350-6

**Published:** 2017-10-28

**Authors:** Daniyal J. Jafree, Amanda C. Williams, Joanna M. Zakrzewska

**Affiliations:** 1 MBPhD Programme, Faculty of Medical Sciences, University College London, Gower Street, London, UK; 2 Research Department of Clinical, Educational and Health Psychology, Faculty of Brain Sciences, University College London, London, UK; 3 Oral Medicine Unit, Eastman Dental Institute, UCLH NHS Foundation Trust, London, UK

**Keywords:** Trigeminal neuralgia, Posterior fossa surgery, PROMs

## Abstract

**Background:**

Microvascular decompression (MVD) and partial sensory rhizotomy (PSR) provide longstanding pain relief in trigeminal neuralgia (TN). Given their invasiveness, complications can result from such posterior fossa procedures, but the impact of these procedures and their complications on patient-reported outcome measures (PROM), such as quality of life and distress, are not well established.

**Method:**

Five years after surgery, patients who underwent first MVD or PSR for TN at one institution, between 1982 and 2002, were sent a self-completion assessment set containing a range of PROMs: the Short Form-12 (SF-12) questionnaire to assess quality of life, the Hospital Anxiety and Depression Scale (HADS) to assess distress, and a questionnaire containing questions about postoperative complications, their severity and impact on quality of life. These findings and demographic data were compared between MVD and PSR.

**Results:**

One hundred and eighty-one of 245 (73.9%) patients after first MVD and 49 of 60 (81.7%) after PSR responded, and were included in analyses. The mean SF-12 scores of patients after MVD and PSR at five-year follow-up were significantly lower than English age-matched norms. Though there were no differences in SF-12 physical or mental component scores between the two procedures, patients after PSR were more likely to have case-level anxiety (RR = 3.3; 95% CI, 1.1–10.5; *p* = 0.03), had more postoperative complications, and of greater severity, including pain (RR = 2.52; 95% CI, 1.5–4.1; *p* < 0.001), numbness (RR = 5.9; 95% CI, 3.8–9.2; *p* < 0.001), burning sensations (RR = 3.0; 95% CI, 1.5–5.8; *p* = 0.001) and difficulty in eating (RR = 17.1; 95% CI, 5.6–53.1; *p* < 0.001), and these had a larger impact on quality of life for PSR compared to MVD.

**Conclusions:**

The quality of life 5 years after MVD or PSR is poorer than in the general population and associated with postoperative complications such as pain, numbness, burning sensation and difficulty in eating. These complications are commoner after PSR than MVD, and this is associated with anxiety in PSR patients at five-year follow-up. However, these differences are not reflected by quality of life scores. Outcome measures need to incorporate patient experience after treatment for TN, and represent patient priorities for quality of life.

**Electronic supplementary material:**

The online version of this article (10.1007/s00701-017-3350-6) contains supplementary material, which is available to authorized users.

## Introduction

Trigeminal neuralgia (TN) presents with severe, unilateral, electric shock-like facial pain provoked by light touch, resulting in significant social disability and associated anxiety and depression [1, 2]. Treatment with anticonvulsant drugs provides some patients with prolonged pain relief and acceptable quality of life. For other patients, the variety of surgical techniques available for TN provide the longest period of pain relief, with a low but significant morbidity and mortality [3–5]. These include invasive posterior fossa procedures, such as microvascular decompression (MVD), partial sensory rhizotomy (PSR), or more recently, internal neurolysis. If neurovascular compression (NVC) is found on magnetic resonance imaging (MRI), MVD is performed, with the aim of decompressing the trigeminal nerve in the region of the root entry zone, where pressure from major arterial vessels is most likely to occur. PSR or internal neurolysis are only carried out if no NVC is found. PSR entails the division of the lateral part of the sensory root, whereas internal neurolysis entails separating nerve fibres longitudinally, and it is postulated that the latter causes less injury to the nerve. PSR, therefore, has become less common [4].

Despite the range of surgical techniques on offer for TN, there are few randomised controlled trials of their efficacy, and existing cohort studies are usually retrospective and of poor quality [6]. These studies do not use standardised outcome measures, making results hard to interpret and compare, resulting in difficulty for patients in choosing between surgical techniques [7, 8]. The primary outcome measure used in many surgical studies for TN is pain relief with or without medication, with neglect of patient-reported outcome measures (PROMs) such as quality of life. Use of PROMS may highlight the potential benefits of posterior fossa procedures for TN over medical management [6]. PROMs to measure pain and its impact have been used in TN, but the impact of other factors on quality of life, such as the fear of return of pain after MVD, or the incidence of numbness after destructive procedures like PSR, is unexplored [9, 10]. A systematic assessment and comparison of the effect of these postoperative complications on quality of life is needed.

### Aims and hypothesis

In this cross-sectional study, we aimed to establish long-term PROMs after first MVD or PSR for TN, including quality of life and distress. Since PSR results in a higher incidence of postoperative complications, while pain relief from MVD and PSR is equivalent [10], we expected to find poorer quality of life and distress in patients undergoing PSR than in patients undergoing their first MVD. We also aimed to determine the relative impact of common postoperative complications on PROMs, 5 years after MVD or PSR.

## Methods

This cross-sectional study is reported in accordance with the STROBE checklist for observational studies [11]. Ethical approval was given by Frenchay Hospital Bristol LREC (Project Number 2001/60).

### Participants and eligibility

Patients were drawn from one neurosurgeon’s entire posterior fossa surgery practice from 1982 to 2002, previously reported in terms of pain recurrence and satisfaction with outcome [12]. The inclusion criteria were: patients with primary idiopathic TN using the diagnostic criteria of International Classification for Headache Disorders [13], and patients who had been followed-up prospectively after MVD or PSR with an operation 6 months or longer before the survey had been carried out. The exclusion criteria were: patients with a secondary cause of TN, clinical evidence of multiple sclerosis, concurrent cranial nerve disorder such as hemifacial spasm, or significant co-morbidities that might influence quality of life or distress.

### Data collection

**Fig. 1:**
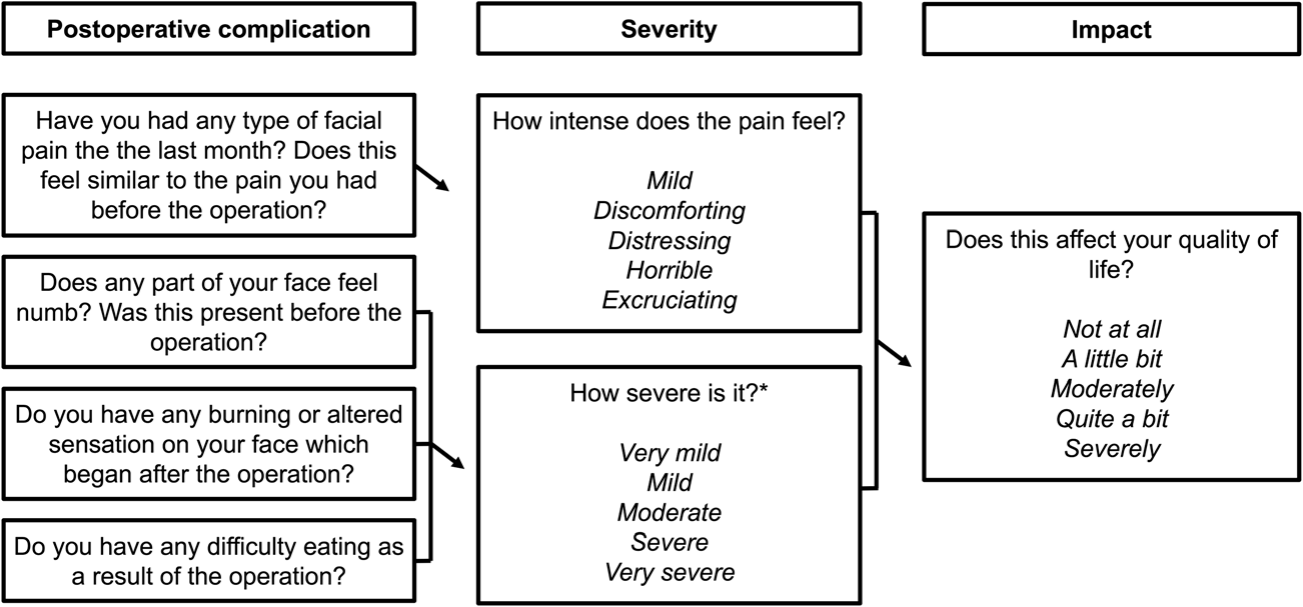
Self-assessment questionnaire. Questions and possible answers provided by the questionnaire as part of the self-assessment set. Severity and impact upon quality of life was asked separately for each postoperative complication. *Possible responses for severity of burning sensation or difficulty in eating differed from severity of numbness, and were: *severe*, *quite a bit*, *moderate* or a *little bit*

Patients were posted a self-completion assessment set relating to PROMs [14]. The Medical Outcomes Study Short Form-12 questionnaire (SF-12) and the Hospital Anxiety and Depression Scale (HADS) were included in the set. Included were questions about the most common postoperative complications, including their presence, severity and impact on quality of life reported by the patient (Fig. 1). Completed assessments were returned to independent observers, including one physician, one neurosurgeon and a patient from the Trigeminal Neuralgia Association UK. For patients who responded, electronic healthcare records were used to provide demographic data including age, gender, co-morbidities, previous surgery, mean duration of symptoms, side of pain, division(s) of the trigeminal nerve affected and current use of medication. The clinical outcomes for this population have already been reported [12]. The main outcome measures for both MVD and PSR patients were PROMs, including: questionnaire results for postoperative complications, quality of life and distress.

The Short-Form 36 (SF-36) and its shorter version, the SF-12, are broad health survey questionnaires used in large studies to assess quality of life across a range of mental and physical health conditions [15]. The SF-12 consists of 12 questions in eight domains: physical functioning, role physical, bodily pain, general health, vitality, social functioning, role emotional and mental health. A raw score for each of the eight domains is calculated, and these scores are weighted and transformed into SF-12 Physical Component Scores (PCS) and Mental Component Scores (MCS), corresponding to the physical and mental dimensions of quality of life, and scored from 0 to 100, where 0 is the lowest possible quality of life, 100 is the highest possible quality of life, and 50 represents the 1998 United States population average. SF-12 scores were calculated as standard [16]. For analyses, SF-12 scores for the MVD and PSR groups were compared with normative data from English 50– to 64-year-olds, collected in 2012 [17]. The HADS is designed for assessment of distress in patients with medical problems, and has been used to evaluate effects of surgery for TN [10, 14, 18]. It is a 14-item scale, with seven items corresponding to anxiety and seven to depression. The response to each item is scored from 0 (absence of symptom) to 3 (high level of symptom), giving scores of 0–21 for anxiety and depression. The cut of point of 8/21 is used for borderline anxiety or depression, and 11/21 for case-level anxiety or depression [19]. The HADS anxiety and HADS depression scores for each patient were then dichotomised either to borderline anxiety and depression or not, and case-level anxiety or depression or not.

### Data analysis

Scores for the SF-12 PCS and MCS were compared for MVD and PSR groups using Mann-Whitney *U* tests. For the HADS, MVD and PSR groups were compared for the numbers of patients with borderline levels or case levels of anxiety or depression using χ^2^ tests. Where demographic factors were significantly different between groups, their effect on the SF-12 and HADS within each group was determined using Mann-Whitney *U* tests and χ^2^ or Fisher’s exact tests respectively. The numbers of patients with postoperative complications, and their severity and impact on quality of life, were compared using χ^2^ tests. Patients with surgery prior to MVD were separated from the cohort and their results reported separately.

All data were managed using Microsoft Excel 2016 (Microsoft, Redmond, VA, USA). Statistical analyses were performed using IBM SPSS Statistics for Macintosh, Version 24.0 (IBM, Armond, NY, USA). Mean values are reported with 95% confidence intervals (95% CI), and median values are reported with interquartile ranges (IQRs). For all statistics, *p* values less than or equal to 0.05 were considered statistically significant. Graphs were drawn using Prism for Macintosh, Version 7 (GraphPad Software, San Diego, CA, USA).

## Results

### Study participants

**Fig. 2:**
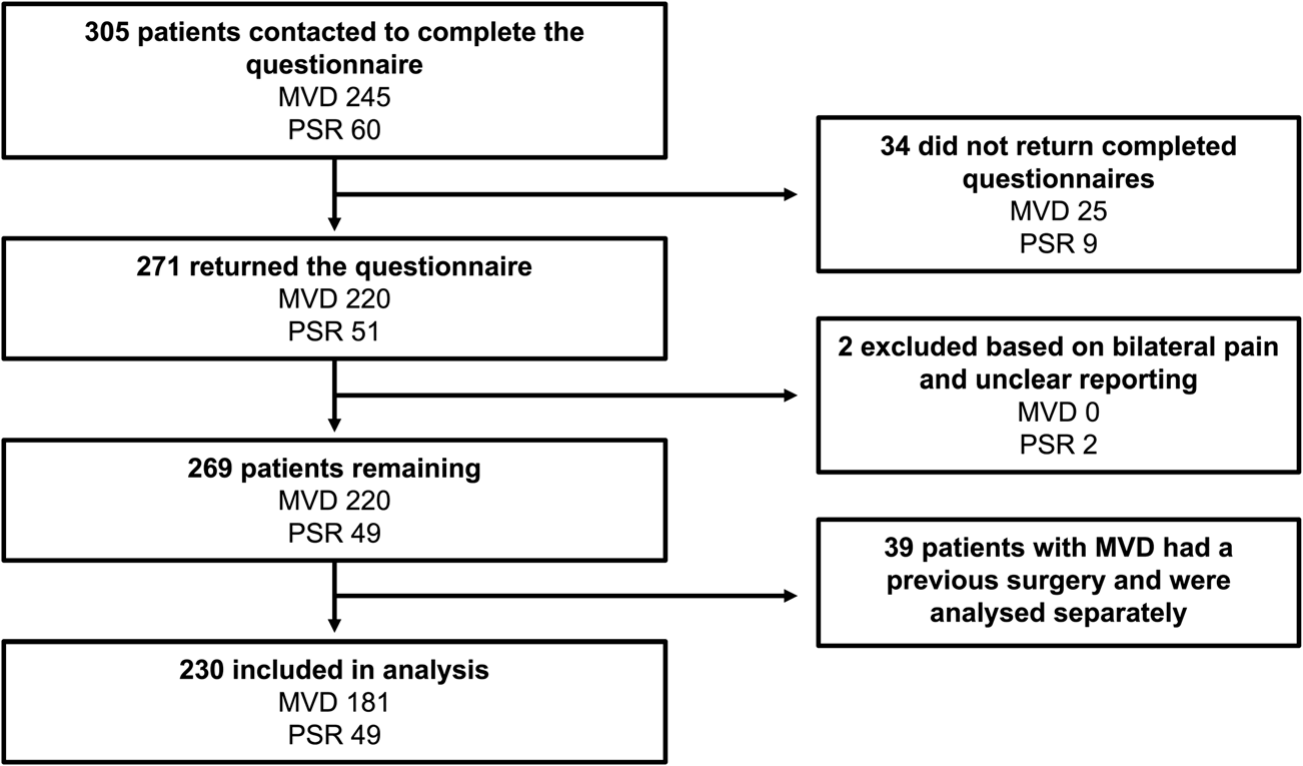
Participant flow diagram

A mean of 5 years after their surgery, 305 patients, of whom 245 underwent MVD and 60 underwent PSR, were contacted and posted self-completion assessment sets (Fig. 2), of whom 271 returned them. Two patients who underwent a PSR experienced bilateral facial pain, and were excluded as the side of pain referred to in the PROMs was unknown. This left 269 (88.2%) from the original cohort. Thirty-nine of 220 (17.7%) responders with an MVD had previous Gasserian ganglion surgery; these were separated from the cohort, leaving 181 patients after MVD (59.2%) and 49 patients after PSR (81.7%) for main analyses.

The characteristics of the 194 patients are described in Table 1. Some variables had missing data in either or both groups, indicated by the smaller denominators. There was a significant difference between the division of the trigeminal nerve affected between MVD and PSR groups (χ^2^ = 23.7; *p* = 0.001). There was also a higher proportion of medication use (χ^2^ = 8.52; *p* = 0.004) in patients who underwent a PSR compared to MVD. Patient co-morbidities were separated into those which were unlikely to have an impact upon quality of life, and those that were, after consensus between a clinician and a psychologist. Of those with co-morbidities, there was a higher proportion of patients with co-morbidities that were likely to impact quality of life after MVD (*n =* 38/73; 52.1%) compared to PSR (*n =* 4/15; 26.7%), but this was not significant (Fisher’s exact test; *p* = 0.092). Nineteen patients out of 49 (38.8%) had previous surgery before PSR. The characteristics of patients with a previous surgery before MVD are reported in Table 2.

**Table 1:** Patient characteristics by group and differences between groups

Patient characteristic	MVD group (*n* = 181)	PSR group (*n* = 49)	Test, *p* value
Mean age in years (± SD)	59.3 (± 11.6)	56.9 (± 12.2)	*t* = 1.24 n.s.
Gender: female (%)	108 (57.9)	34 (69.4)	χ^2^ = 1.54 n.s.
Other co-morbidities (%)	78 (43.1)	16 (32.7)	χ^2^ = 1.74 n.s.
Median duration of symptoms in years (IQR)	5.0 (2.5–8.0)	4.0 (2.0–9.0)	*U =* 2,802.0 n.s.
Side of pain (%): right, left	116/172 (67.4), 56/172 (32.6)	27 (55.1), 22 (44.9)	χ^2^ = 2.54 n.s.
Division affected (%)			χ^2^ = 23.7 *p* = 0.001
V1	11/164 (6.7)	0/46 (0.0)	
V2	20/164 (12.2)	10/46 (21.7)	
V3	36/164 (22.0)	10/46 (21.7)	
V1 + V2	27/164 (16.5)	0/46 (0.0)	
V2 + V3	13/164 (7.9)	1/46 (2.2)	
V1 + V2 + V3	57/164 (34.8)	25/46 (54.3)	
Currently taking medication for pain (%)	14/180 (7.8)	11/49 (22.4)	χ^2^ = 8.52 *p* = 0.004
Occasional use	2/14 (14.2)	2/10 (20.0)	
Once daily use	9/14 (64.3)	6/10 (60.0)	
Use twice daily or more	3/14 (21.4)	2/10 (20.0)

**Table 2:** Patient characteristics of MVD patients with previous surgery

Patient characteristic	MVD group with previous surgery (*n* = 39)
Mean age in years (± SD)	59.3 (± 11.6)
Gender: female (%)	23 (59.0)
Other co-morbidities (%)	19 (48.9)
Median duration of symptoms in years (IQR)	5.0 (2.5–8.0)
Side of pain (%): right, left	22 (56.4), 17 (43.6)
Division affected (%)	
V1	4/37 (10.8)
V2	6/37 (16.2)
V3	11/37 (29.7)
V1 + V2	6/37 (16.2)
V2 + V3	2/37 (5.4)
V1 + V2 + V3	8/37 (21.6)
Currently taking medication for pain (%)	10 (25.6)
Occasional use	1/10 (10.0)
Once daily use	9/10 (90.0)

### PROMs after MVD or PSR

**Fig. 3:**
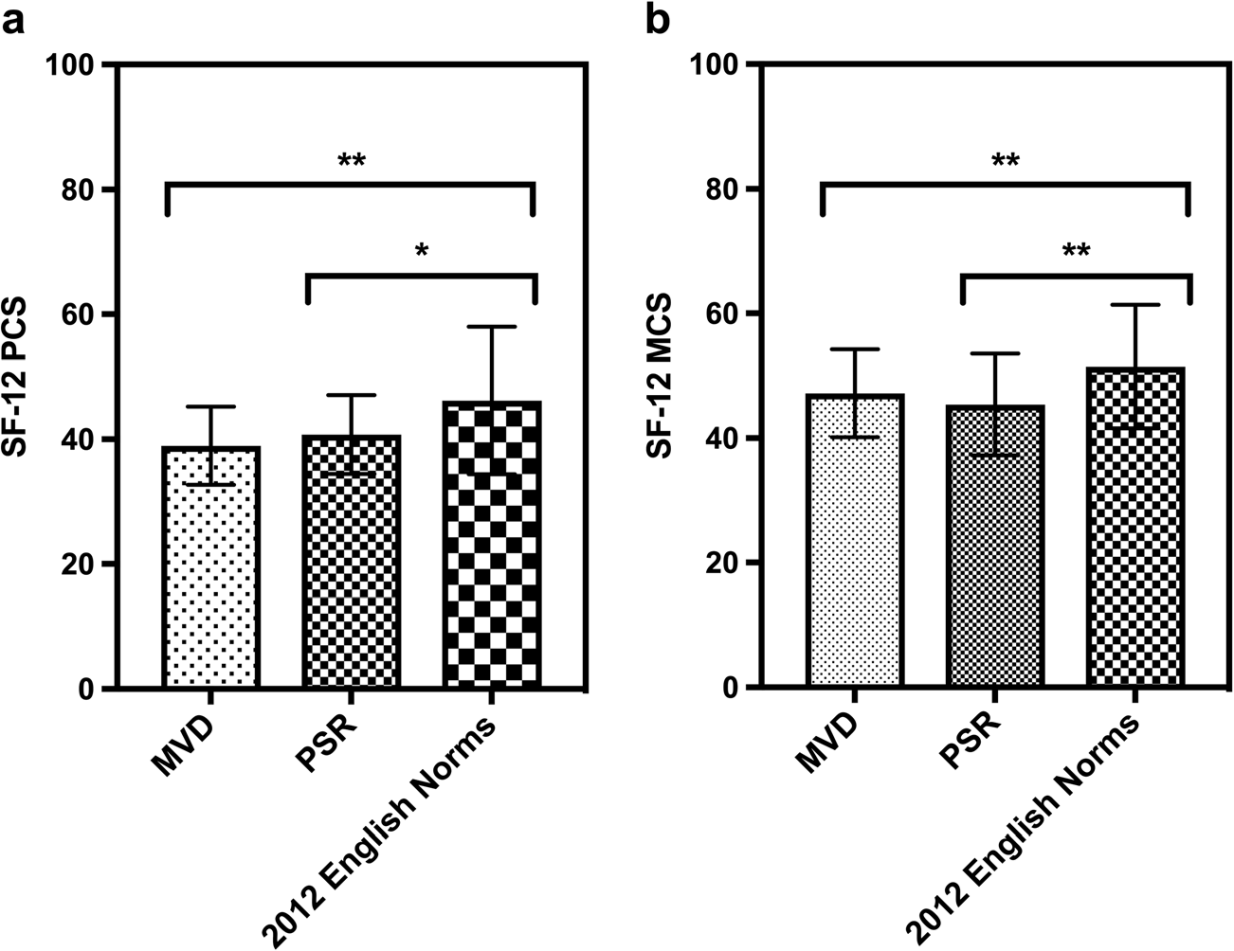
Comparison of SF-12 PCS and MCS with UK norms from 2012. The mean SF-12 PCS (**a**) and MCS (**b**) scores of MVD and PSR patients and those of English norms of 50– to 64-year-olds in 2012 were compared using ANOVA and Tukey’s pairwise multiple comparisons. *Error bars* represent standard deviations; significance levels: * *p <* 0.05, ** *p <* 0.01

The SF-12 and HADS were used to compare patients 5 years after MVD or PSR (Supplementary Material 1). There were no significant differences between the median SF-12 physical component score or median mental component score of patients in the two groups, but mean SF-12 scores for both were significantly lower than contemporary English norms for 50– to 64-year-olds (*n* = 2,214): mean PCS, 46.2 (SD, 11.8) and mean MCS 51.5 (SD, 9.9) (Fig. 3). ANOVA with Tukey’s pairwise comparisons showed significantly lower PCS and MCS scores after MVD (F = 33.6; *p* < 0.0001 for both PCS and MCS) or PSR (F = 22.1; *p* < 0.0063 for PCS and *p =* 0.0002 for MCS). Using χ^2^ tests, it was found that patients after PSR were more likely to have case-level anxiety, scoring 11/21 on the HADS, than after MVD (relative risk; RR = 3.3; 95% CI, 1.1–10.5; χ^2^ = 4.71; *p* = 0.03).

Of 39 patients with previous surgery after MVD, 31 completed the SF-12. Their median SF-12 PCS was 39.2 (IQR, 36.2–44.3) and median SF-12 MCS was 45.0 (IQR, 41.3–51.3). Thirty-eight of these patients completed the HADS, of whom 14 (36.8%) demonstrated borderline anxiety on the HADS, five (13.2%) case-level anxiety, nine (23.7%) borderline depression and three (8.9%) case-level depression. To determine the effect on PROMs of the differences in characteristics between groups, division of the trigeminal nerve affected and medication use were compared by PROMs within the MVD and PSR groups using Mann-Whitney *U* tests and contingency analyses. In the MVD group, patients on regular pain medication were more likely to have borderline depression (Fisher’s exact test; *p =* 0.043), whereas in the PSR group, medication use was also associated with case-level depression (Fisher’s exact test; *p =* 0.008). Also in the PSR group, the median SF-12 MCS of those on medication at follow-up (median, 39.1; IQR, 28.6–49.7) was significantly lower than that of those not taking medication (median, 49.4; IQR, 42.8–50.9; *U* = 81.0; *p* = 0.02).

### PROMs by postoperative complication after MVD or PSR

The most common postoperative complications were pain, numbness, burning sensations and difficulty in eating. A comparison of the two surgical procedures in terms of postoperative complications was made (Table 3). Significantly greater proportions of patients experienced pain (RR = 2.52; 95% CI, 1.5–4.1), numbness (RR = 5.9; 95% CI, 3.8–9.2), burning sensation (RR = 3.0; 95% CI, 1.5–5.8) and difficulty in eating (RR = 17.1; 95% CI, 5.6–53.1) after PSR than after MVD. Patients directly rated the impact of each postoperative complication on their quality of life (Fig. 1). For both MVD and PSR, the severity rating for each postoperative complication correlated with the rating of impact on quality of life (*p* < 0.001). Data for the impact of pain on quality of life was not available. PSR patients reported greater impact on quality of life of numbness (χ^2^ = 81.1; *p <* 0.001), burning (χ^2^ = 26.0; *p <* 0.001) and difficulty in eating (χ^2^ = 39.5; *p <* 0.001) than MVD patients (Table 4). The presence of postoperative complications, their severity and impact on quality of life in patients who had surgery prior to their MVD are reported in Table 5.

**Table 3:** Postoperative complications and severity after MVD or PSR

Postoperative complication	MVD group	PSR group	Test, *p* value
Pain (%)	25/157 (15.9)	18/42 (42.9)	χ^2^ = 12.4, *p* < 0.001
Excruciating	2/25 (8.0)	1/18 (5.6)	
Horrible	5/25 (20.0)	3/18 (16.7)	
Distressing	7/25 (28.0)	2/18 (11.1)	
Discomforting	5/25 (20.0)	10/18 (55.6)	
Mild	6/25 (24.0)	2/18 (11.1)	
Numbness (%)	20/157 (14.6)	6/48 (75.0)	χ^2^ = 71.7, *p* < 0.001
Very severe	2/20 (10.0)	3/36 (8.3)	
Severe	1/20 (5.0)	9/36 (25.0)	
Moderate	5/20 (25.0)	10/36 (27.8)	
Mild	5/20 (25.0)	8/36 (22.2)	
Very mild	7/20 (35.0)	6/36 (16.7)	
Burning (%)	14/157 (8.9)	13/49 (26.5)	χ^2^ = 10.2, *p* = 0.0014
Severe	0/14 (0.0)	2/13 (15.4)	
Quite a bit	2/14 (14.3)	5/13 (38.5)	
Moderate	1/14 (7.1)	2/13 (15.4)	
A little bit	11/14 (78.6)	4/13 (30.8)	
Difficulty in eating (%)	3/157 (1.9)	16/49 (32.7)	Fisher’s test, *p* < 0.001
Severe	0/3 (0.0)	3/16 (18.8)	
Quite a bit	0/3 (0.0)	2/16 (12.5)	
Moderate	1/3 (33.3)	5/16 (31.3)	
A little bit	2/3 (66.7)	6/16 (37.5)

**Table 4:** Impact of postoperative complications on quality of life between MVD and PSR

Impact of postoperative complications upon quality of life	MVD group	PSR group	Test, *p* value
Impact of numbness on quality of life (%)			χ^2^ = 81.1, *p* < 0.001
Severe	0/20 (0.0)	2/36 (5.6)	
Quite a bit	0/20 (0.0)	5/36 (13.9)	
Moderately	2/20 (10.0)	7/36 (19.4)	
A little bit	6/20 (30.0)	11/36 (30.6)	
Not at all	12/20 (60.0)	11/36 (30.6)	
Impact of burning on quality of life (%)			χ^2^ = 26.0, *p* < 0.001
Severe	0/15 (0.0)	3/15 (20.0)	
Quite a bit	0/15 (0.0)	2/15 (13.3)	
Moderately	1/15 (6.7)	4/15 (26.7)	
A little bit	5/15 (33.3)	4/15 (26.7)	
Not at all	9/15 (60.0)	2/15 (13.3)	
Impact of difficulty in eating on quality of life (%)			χ^2^ = 39.5, *p* < 0.001
Severe	0/7 (0.0)	3/17 (17.6)	
Quite a bit	0/7 (0.0)	2/17 (11.8)	
Moderately	0/7 (0.0)	2/17 (11.8)	
A little bit	3/7 (42.9)	7/17 (41.2)	
Not at all	4/7 (57.1)	3/17 (17.6)

**Table 5:** Postoperative complications after MVD with prior surgery

Postoperative complication	Severity	Impact on quality of life
Pain (%)	17/39 (43.6)	
	Excruciating 3/17 (17.6)	–
	Horrible 2/17 (11.8)	–
	Distressing 3/17 (17.6)	–
	Discomforting 5/17 (29.4)	–
	Mild 4/17 (23.5)	–
Numbness (%)	8/28 (28.6)	
	Very severe 0/8 (0.0)	Severe 0/9 (0.0)
	Severe 1/8 (12.5)	Quite a bit 2/9 (22.2)
	Moderate 3/8 (37.5)	Moderately 0/9 (0.0)
	Mild 0/8 (0.0)	A little bit 3/9 (33.3)
	Very mild 4/8 (50.0)	Not at all 4/9 (44.4)
Burning (%)	4/29 (13.8)	
	Severe 2/4 (50.0)	Severe 1/5 (20.0)
	Quite a bit 0/4 (0.0)	Quite a bit 1/5 (20.0)
	Moderate 0/4 (0.0)	Moderately 0/5 (0.0)
	A little bit 2/4 (50.0)	A little bit 1/5 (20.0)
	–	Not at all 2/5 (40.0)
Difficulty in eating (%)	3/29 (10.3)	
	Severe 1/3 (33.3)	Severe 1/4 (25.0)
	Quite a bit 0/3 (0.0)	Quite a bit 1/4 (25.0)
	Moderate 1/3 (33.3)	Moderately 0/4 (0.0)
	A little bit 1/3 (33.3)	A little bit 0/4 (0.0)
	–	Not at all 2/4 (50.0)

## Discussion

This prospective study assessed all patients with TN a mean 5 years after MVD or PSR using a range of PROMs, including SF-12, the HADS and questions about postoperative complications. The results varied according to the PROM used. Patients’ responses supported our main hypothesis, that PSR, given its higher rate and severity of complications [12], is associated with poorer quality of life and that pain recurrence is not the only factor impacting on quality of life. The SF-12 showed that the physical and mental dimensions of quality of life were lower in patients 5 years after posterior fossa surgery than UK norms from 2012. The SF-12 did not significantly differ between MVD and PSR, though patients were over 3 times more likely to have case-level anxiety on the HADS after PSR than after MVD.

This is the first study to prospectively compare long-term PROMs between two posterior fossa procedures for the treatment of TN. Survey and data collection were carried out by a physician, neurosurgeon and patient, all of whom were independent of the surgical unit. Patients with co-morbidities unrelated to facial pain were also included, ensuring the cohort was as representative of the TN population as possible. However, our study is not without limitations. As PSR is less commonly performed than MVD, the number of patients in the PSR group is relatively small, and so the comparison between the two procedures should be interpreted with caution. Though the four most common postoperative complications are reported in this cohort, this does not accommodate for other complications, such as hearing loss, headaches, dizziness or visual problems, which may impact PROMs and account for the low SF-12 scores in both surgical groups [20, 21]. The HADS has come under increasing criticism for an unstable structure that affects interpretability [22]. The decision to score anxiety and depression separately and categorically was taken before these problems became clear, but made patient distress hard to interpret. Not all patients completed every item of the SF-12, which means for 22 patients after MVD and seven patients after PSR, the SF-12 PCS and MCS could not be calculated.

Other studies have also compared MVD and PSR, but comparison with these is difficult to make due to their focus on pain relief with or without presence of complications [23–29]. Some studies found that postoperative complications are more frequent after PSR than MVD, but others found that the destructive procedure yields similar pain outcomes and equivocal postoperative complications. Our results suggest that patients after PSR are more anxious and have more frequent postoperative complications, which have a greater severity and impact on quality of life than MVD. The high incidence of complications and numbness in this cohort is consistent with previous reports of PSR [30]. As demonstrated by patient responses, postoperative complications such as numbness and burning can seriously affect quality of life, and this is also reported after stereotactic radiosurgery (SRS) [31]. Though these complications are well reported after destructive procedures, their presence and frequency after MVD in our cohort was surprising. It was demonstrated that 14.6% of patients undergoing MVD may develop numbness, which is milder than after PSR, but can impact on quality of life. Previous studies have shown neurosensory disturbance after MVD, including numbness and change of taste [20, 21]. Only one study in a cohort of patients used the Japanese version of the SF-36 to assess quality of life after MVD [32]. In this study, the individual norm-based items: physical role, emotional role and social functioning were significantly lower than the Japanese population 2 years after MVD, similar to our comparison with the UK population sample. The only other two studies assessing quality of life after surgery for TN reported individual norm-based items of the SF-36 after SRS. Azar and colleagues [33] found improved SF-36 scores after SRS, with a mean follow-up of 54 months. Pan and colleagues [34] found higher scores for individual items of the SF-36 to be associated with lower pain severity, but did not report on other complications. Comparison to these studies is difficult, because of their use of individual items of the SF-36, rather than calculation of PCS and MCS.

Posterior fossa procedures aim to provide immediate pain relief for TN. MVD is considered the procedure of choice for classic TN with NVC, but in patients without NVC, with TN relapsing after MVD or in patients with a vascular anatomy that prohibits safe decompression, destructive procedures such as PSR have been considered a safe and effective alternative [30]. Although PSR has largely been discontinued in routine clinical practice, internal neurolysis is now advocated in those with no NVC but, as with PSR, the incidence of numbness after internal neurolysis is high [4]. Neurosensory disturbance of the trigeminal nerve results in poorer health-related quality of life [35], as found in our study. There have been no head-to-head comparisons of PSR against internal neurolysis, but as they are both destructive procedures, surgeons must warn patients of the increased risk of complications, such as numbness, and their impact on anxiety and wellbeing to ensure informed consent has been obtained. The difference between SF-12 scores of patients and English norms from 2012 is concerning. This may be due to postoperative complications and recurrence of pain, but may also be due to fear of the return of pain, which was not assessed in this study. In TN, there is risk of development of depressive, anxiety or sleep disorders [1], which also reduces quality of life compared to the general population, and there is no evidence to suggest that posterior fossa surgery alleviates these. Regardless of the reasons for poorer quality of life in these patients, these results suggest unmet needs after surgery, and in this respect and as previously suggested, that the current management of TN is suboptimal [36]. Future studies should seek to determine whether posterior fossa surgery reduces the incidence of anxiety, depression and fear of recurrence in TN more effectively than other modalities, such as medications and Gasserian ganglion surgery. In this cohort, though the patients’ responses revealed PSR to yield more common and severe complications that impact quality of life, this was not reflected by the SF-12. This may be because the SF-12 provides a generalised measure of the physical and mental dimensions of quality of life, and is not sensitive to the impact of these postoperative complications after surgery for TN. Therefore, the next step is to determine from patients themselves what outcomes they consider important, often divergent from those routinely assessed, and to estimate minimally important clinical differences, as has been done for SRS patients [37, 38]. A recent review identified over 116 quality of life scales that were categorised into 32 health domains [39]. Ideally a quality of life scale not only represents patients’ priorities but can also be translated into quality-adjusted life years, which in turn, can be represented in economic terms to guide treatment choices [40]. As TN is managed by a wide variety of medical and surgical interventions, arriving at a consensus for what constitutes the best quality of service would be of considerable value, as has been done for headaches [41].

## Conclusions

In conclusion, PSR was associated with a higher risk of postoperative complications than MVD, which was associated with anxiety and impacted patients’ rated quality of life, although this was not detected by the SF-12. The quality of life of postoperative patients with TN is lower than that of the age-matched English population, regardless of the surgical procedure, suggesting that this disorder should be regarded as a long-term condition. Patient-centred outcomes and their assessment in TN remains an under-researched area, but one of considerable clinical and economic importance.

## Electronic supplementary material

ESM 1 (DOCX 60 kb)

## Abbreviations

HADS: Hospital Anxiety and Depression Scale
MCS: mental component score
NVC: neurovascular compression
PCS: physical component score
PROM: patient-reported outcome measure
PSR: partial sensory rhizotomy
RR: relative risk
TN: trigeminal neuralgia
SRS: stereotactic radiosurgery
