# Supplementary material for: Impact of pain and postoperative complications on patient-reported outcome measures 5 years after microvascular decompression or partial sensory rhizotomy for trigeminal neuralgia
Source: Acta Neurochir (Wien). 2017 Oct 28;160(1):125–34. doi: 10.1007/s00701-017-3350-6 (PMC5735194; doi:10.1007/s00701-017-3350-6)
Supplement: Supplementary file 1 — (DOCX 60 kb) [file 701_2017_3350_MOESM1_ESM.docx]

**Impact of pain and post-operative complications on patient-reported outcome measures five years after posterior fossa surgery for trigeminal neuralgia**

Daniyal J Jafree*, ^1^ Amanda C Williams, ^2^ Joanna M Zakrzewska ^3^

^1^ *MBPhD Programme, Faculty of Medical Sciences, University College London, London, UK*

^2^ *Research Department of Clinical, Educational and Health Psychology, Faculty of Brain Sciences, University College London, London, UK*

^3^ *Oral Medicine Unit, Eastman Dental Institute, UCLH NHS Foundation Trust, London, UK*

***: Corresponding author details:**

Daniyal J Jafree

MBPhD Programme,

Faculty of Medical Sciences,

University College London,

Gower Street,

London, WC1E 6BT

Telephone: +44(0)7748417402

Email: [daniyal.jafree.13@ucl.ac.uk](mailto:daniyal.jafree.13@ucl.ac.uk)

**Article type:** Original research

**Supplementary Material 1 | SF-12 and HADS results 5 years after MVD or PSR**

| **Outcome** | **MVD group** | **PSR group** | **Test, *p* value** |
| --- | --- | --- | --- |
| Median SF-12 score (IQR)* |  |  |  |
| *SF-12 physical component* | *39.4 (35.2-42.3)* | *39.4 (37.2-44.3)* | *U = 3747, ns* |
| *SF-12 mental component* | *47.9 (43.7-50.9)* | *47.4 (41.4-50.9)* | *U = 3107, ns* |
| HADS (%) |  |  |  |
| *Borderline anxiety* | *36/163 (22.1)* | *14/46 (30.4)* | *χ^2^ = 1.37, ns* |
| *Case-level anxiety* | *7/163 (4.3)* | *6/46 (13.0)* | *χ^2^ = 4.71, p = 0.03* |
| *Borderline depression* | *20/164 (12.2)* | *9/46 (19.6)* | *χ^2^ = 1.64, ns* |
| *Case-level depression* | *4/164 (2.4)* | *3/46 (6.5)* | *χ^2^ = 1.86, ns* |

*Due to incomplete questionnaires, the SF-12 results of 159 MVD patients and 42 PSR patients are shown
